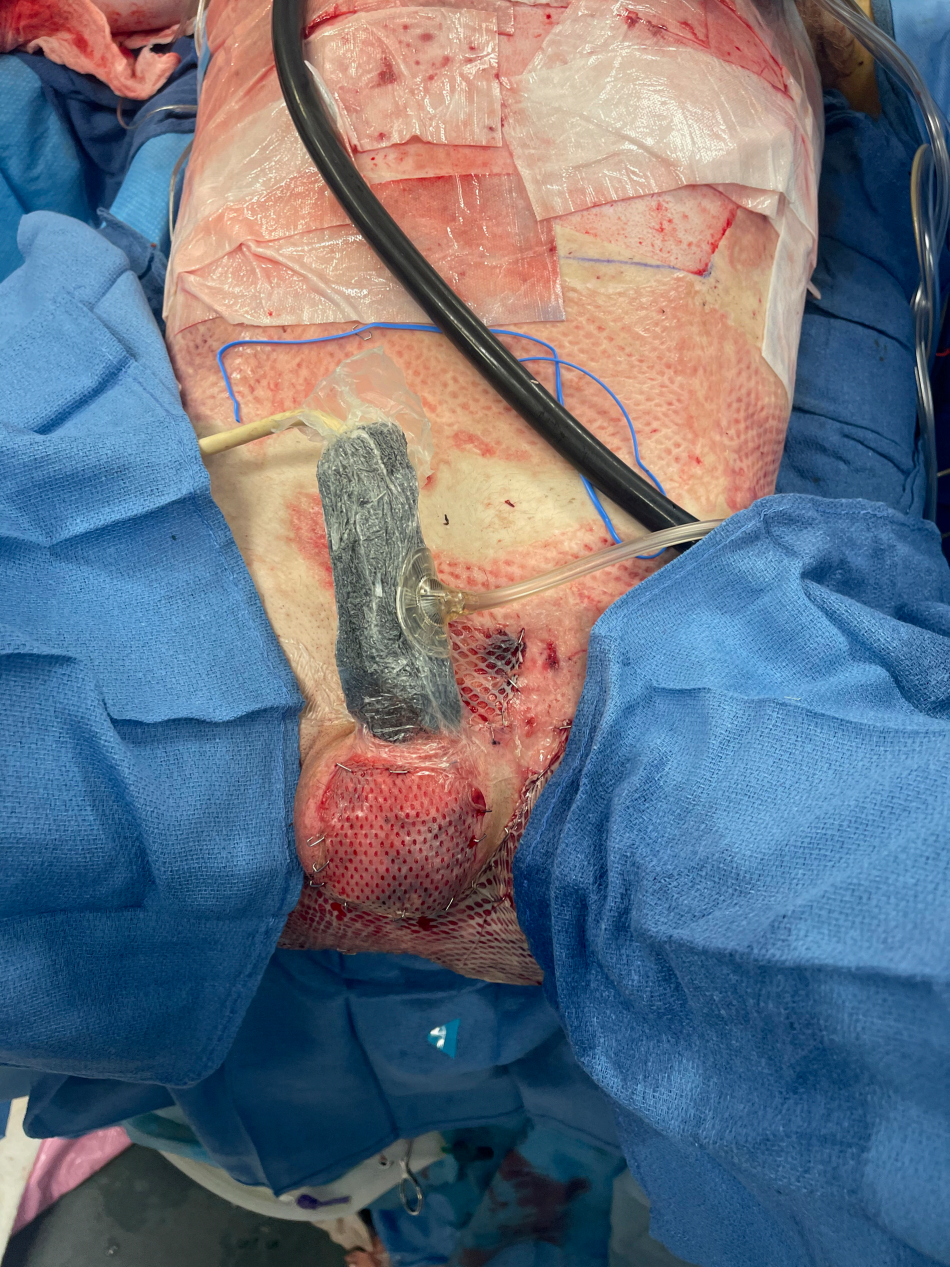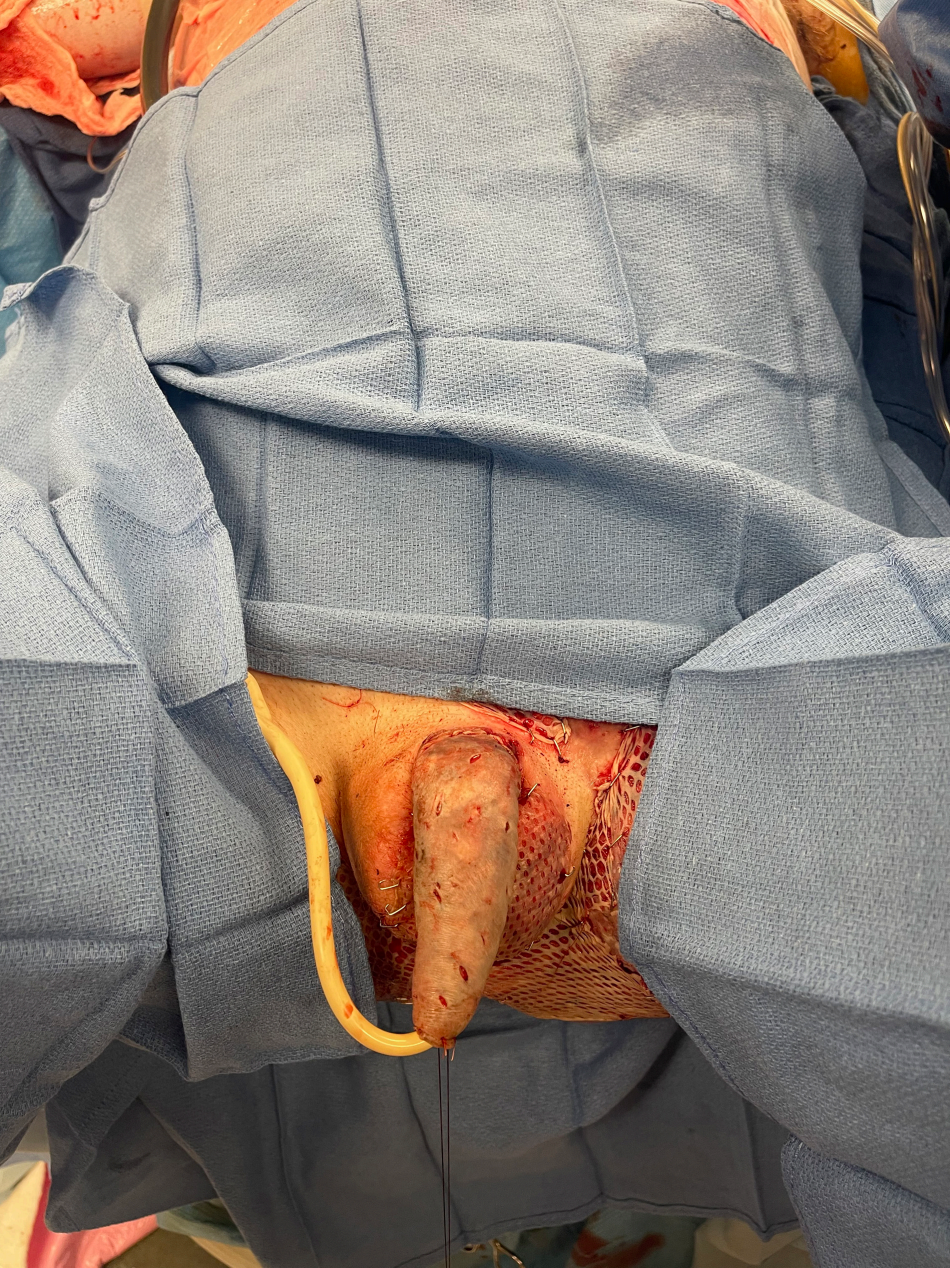# 856 Summary of Case Study: Management of Full Thickness Genital Burns in an 18-Year-Old Male

**DOI:** 10.1093/jbcr/iraf019.387

**Published:** 2025-04-01

**Authors:** Denise Knight, David Hill, Mahmoud Hassouba

**Affiliations:** Firefighters Regional One Burn Center; Firefighters Regional One Burn Center; University of Tennessee Health Science Center

## Abstract

**Introduction:**

This case study addresses the complex surgical management of full thickness genital burns, focusing on maintaining urogenital function, preventing scar contracture, and achieving satisfactory aesthetic outcomes. An 18-year-old male sustained 43.5% total body surface area (TBSA) burns from an electrical arc incident at work, affecting his abdomen, torso, and genital regions.

**Methods:**

The treatment involved multiple surgical interventions in staged reconstruction, including burn wound excision and graft applications over several weeks. Key procedures included:

Day 2: Initial excision and allograft application to lower extremities, abdomen, penis, and scrotum.

Day 8: Additional excisions and allograft application to penis and scrotum.

Day 11: Repeat excision with application of synthetic matrix skin substitute to his perineum, scrotum, and penis.

Day 39: Final operation involving intraoperative artificial erection to maximize penile length, followed by a 1:1 autograft to scrotum and sheet graft to penis with immediate splinted negative pressure therapy to prevent contracture during early post-operative healing.

The patient gave consent for the purpose of this case study.

**Results:**

The patient achieved 100% scrotal and 99% penile graft adherence on evaluation after two days of negative pressure therapy. At the nine-month follow-up, he reported non-painful erections. However, his hospital course was complicated by a cefepime-resistant Pseudomonas aeruginosa urinary tract infection, multi-organism bacteremia, and colonic dilation due to ileus.

**Conclusions:**

This case illustrates the significant surgical challenges in managing full thickness genital burns, emphasizing the necessity for tailored approaches in both intraoperative and postoperative care. The use of negative pressure therapy and diverse grafting techniques contributed to positive outcomes, despite the complications encountered.

**Applicability of Research to Practice:**

The study underscores the importance of careful monitoring and innovative strategies in the management of such complex burn injuries.

**Funding for the Study:**

N/A